# Malnutrition in School-Going Adolescents in Dessie Town, South Wollo, Ethiopia

**DOI:** 10.1155/2021/4898970

**Published:** 2021-01-07

**Authors:** Gizachew Mulu Birru, Sisay Eshete Tadesse, Kalkidan Hassen Abate, Tefera Chane Mekonnen, Muluken Genetu Chane

**Affiliations:** ^1^Care Ethiopia, Hawassa Field Office, Hawassa, Ethiopia; ^2^Researchers and Academician at School of Public Health, College of Medicine and Health Sciences, Wollo University, Dessie, Ethiopia; ^3^Department of Nutrition and Dietetics, Faculty of Public Health, Jimma University, Jimma, Ethiopia

## Abstract

**Background:**

Malnutrition among adolescents is a global public health problem. Nutrient intake is a proxy measure of nutritional status, but studies in developing countries describing the actual nutrient intake condition associated with nutrition in the adolescents are limited. On top of this, there is inconsistent finding on the extent of malnutrition among adolescents. Hence, the aim of this study was to assess malnutrition and the associated factors among adolescents at Dessie high school.

**Methods:**

A school-based cross-sectional study was conducted among 365 randomly selected high school adolescents. The data were collected using a structured questionnaire adapted from previous literature studies. Nutrient intakes were estimated by ESHA food processor software from a 24-hour recall, and anthropometric indices were calculated from weight, height, age, and sex. The data analysis was managed by SPSS version 23. Binary logistic regression and multinomial logistic regression were used to report the associated factors of malnutrition. Adjusted odds ratio with 95% CI was used to reveal the presence of statistical association.

**Results:**

The percentage of being stunted, underweight, and overweight/obese was 15.7%, 6.3%, and 8.2%, respectively. Snack consumption (AOR = 0.38, 95% CI: 0.20, 0.71) was negatively associated with stunting, while MAR <1 (AOR = 3.36, 95% CI: 1.15, 7.82) was positively associated with stunting. Being a male (AOR = 2.76, 95% CI: 1.03, 7.44) and meal consumption <3 times per day (AOR = 4.21, 95% CI: 1.35, 13.11) were factors positively associated with being underweight/thin. Dietary diversity score <5 (AOR = 0.35, 95% CI: 0.13, 0.89) was negatively associated with overweight/obesity, while MAR < 1 (AOR = 3.14, 95% CI: 1.09, 9.09) was positively associated with overweight/obesity.

**Conclusion:**

The percent of overweight/obesity among adolescents in the study area was higher compared with the national and regional prevalence, and this was found to be a public health concern. Therefore, increasing snack consumption, sex consideration, increasing meal consumption, and intake of diversified foods should be included in the prevention strategies of malnutrition among adolescents.

## 1. Introduction

Malnutrition in adolescent encompasses both under- and overnutrition, which include underweight, stunting and micronutrient deficiencies, and overweight for one's age and sex [1, 2]. The dual burden of malnutrition in the same adolescent reflects poor quality of diet and morbidity in the first two years of life, followed by excess energy consumption at a later life stage [3]. Adolescents face a serious nutritional challenge that would affect their rapid growth spurt as well as their health [4]. Inadequate diet during this period can result in a decreased learning ability, delayed sexual maturity, micronutrient deficiency, and lack of concentration, impaired school performance, undermining physical and economic growth, limiting the body's ability to appropriately absorb nutrients, and perpetuating poverty [5].

Worldwide, 10% of adolescents are overweight and 2–3% are obese. The percent of adolescent girls and boys aged 15–19 years who are thin is 29% and 59%, respectively [4]. Overweight/obesity is now the fifth leading risk for mortality worldwide [6]. According to World Health Organization (WHO) estimates, noncommunicable chronic diseases will account for approximately three quarters of all deaths in the developing world by the year 2020 [7]. Malnutrition in adolescence is associated with deficiencies in muscular strength, working capacities, poor educational performance, easy susceptibility to infection, increase of the risk of chronic noncommunicable diseases, disability, and kicking off the intergenerational effect of malnutrition [4, 8–10].

Previous findings showed that family income, number of meals consumed per day, meal skipping pattern, diet diversity, family size, illness, living style, genetics, place of residence, parents' culture, and economic assets of the family plays a role in the development of malnutrition among adolescents [11, 12]. Overweight/obesity is mainly associated with economic growth and urbanization [13].

Despite the efforts made by the Ethiopian government and stakeholders' malnutrition is a public health problem among Ethiopian adolescents. Nutrient intake is a proxy measure of nutritional status, but studies in developing countries are limited in describing the actual nutrient intake condition with adolescent nutrition. On top of this, there are inconsistent findings on the extent of malnutrition among adolescents [8, 9, 14]. Investing in adolescent health yields triple dividends, that is. improved health for the adolescents now, productive force for tomorrow's economy, and healthy parents that can reproduce and nurture their children. It is the second opportunity period in breaking the vicious cycles of malnutrition [15] and helping a stunted child or adolescent undergo a catch-up growth period and resume a normal growth trajectory before his/her height is permanently reduced. Particularly in the study area, there is no published study on malnutrition among adolescents. Therefore, the aim of this study was to fill the identified gaps by assessing the malnutrition and associated factors among high school adolescents at Dessie town. The results of this study could be useful for health managers and program planners when designing prevention strategies related to overweight/obesity among adolescent in the country with a similar context.

## 2. Methods and Materials

### 2.1. Study Setting

A school-based cross-sectional study design was conducted from April 16 to April 27, 2018, in Dessie town, South Wollo Zone. It is located 401 km far from Addis Ababa and 490 km from Bahir Dar. It is found at an altitude between 2,470 and 2,550 meters above sea level. According to 2017 population projection of Ethiopia, the town has a total population of 209,226 of whom 103,429 (49.4%) were men and 105,797 (50.6%) were women. The town is subdivided into five subcities. There are 13 high schools (9 governmental schools and 4 private schools) with a total of 9,051 students in grades 9 and 10. The number of students from governmental and private schools was 712 and 8,339, respectively.

### 2.2. Source and Study Population

All adolescents attending education in all high schools of Dessie town were the source population, while adolescents who attended their education in the selected high schools were the study population.

### 2.3. Inclusion and Exclusion Criteria

All adolescents attending education in the selected high schools were included in the study. However, adolescents with physical deformity, pregnant, and with severe illness were excluded from the study.

### 2.4. Sample Size Determination and Sampling Procedures

The sample size was calculated using single population proportion formula by considering the proportion of stunting among adolescents taken from the regional finding 31.5% [16], confidence level of 95%, and 10% marginal error. The final sample size was 365.

### 2.5. Sampling Procedures

From 13 high schools, four high schools were randomly selected and included in the study. The sample size was distributed proportionally to each selected high school based on their total number of students by considering the ratio of girls and boys. Random number was generated from class roster of students' using their identification number, and study participants were included in the study by simple random sampling technique.

### 2.6. Data Collection Tool and Measurement

Data were collected using a structured questionnaire, weight-measuring scale, height-measuring board, and 24 hours' recall with face-to-face interview. Three BSc nurses and one supervisor were recruited to collect the data. Adolescents' height was measured using a standard height measuring board (stadiometer) and recorded to the nearest 0.1 centimeters. Weight was measured by SECA Germany weight measuring scale to the nearest 0.1 Kg. A twenty-four hour recall was used to assess the dietary diversity score and amount of nutrients consumed within 24 hours, and then the adequacy of the nutrients was compared with the standard value of the recommended daily allowance.

The study variables included in the study were the nutritional status of adolescents, sociodemographic features (religion, sex, age, fathers'/mothers' educational status, school type, grade level, fathers'/mothers' occupation, family monthly income, marital status of parents, ethnicity, family size, and sex of household heads), dietary- and life-style-related factors (meal frequency, food items consumed, snack, breakfast skipping, staple cereal, vitamin-A-rich food consumption, micronutrient supplementation, deworming, nutrition counseling, school feeding, adequacy of nutrient consumption (NAR & MAR), alcohol consumption, physical activity (GPAQ), and means of transportation), and WASH-related factors (type of drinking water, availability and utilization of latrine, and hand-washing practice).

### 2.7. Data Quality Assurance

The questionnaire was translated to Amharic (local language) and back to English to check the consistency. Then, the tool was pretested on 5% (19 students) of the sample at Kombolcha high school; data collectors and a supervisor were trained for two days. To minimize measurement errors, calibration was made using anthropometric measurement scales. Two measurements were taken and the average was used.

### 2.8. Data Analysis

After coding, data were entered into Epi Data version 3.1. Then, the data were transferred to SPSS version 23, ESHA food processor, and WHO 2005 Anthroplus software for analyses. Data cleaning was performed by simple frequency and cross-tabulation. The dietary diversity was determined by the number of foods consumed from 10 food groups within 24 hours. Then, dietary diversity score was categorized as poor (<5 food groups) and good dietary diversity (≥5). Anthropometric indices were generated by WHO Anthroplus software to determine BMI for age and HAZ score. Nutrient adequacy ratio and mean adequacy ratio were generated by ESHA food processer software. Descriptive statistics was done and reported using frequency, mean (standard deviation), graphs, percentages, and tables. The binary logistic regression model was used to identify factors associated with stunting. While multinomial logistic regression was computed to determine factors associated with underweight/thinness and overweight/obesity. In both models, variables having a *p*-value<0.25 in bivariable regression were transported to multivariable regression model. In the final model, adjusted odds ratio with 95% confidence interval and a *p*-value <0.05 were used to identify factors associated with malnutrition among adolescents. Multicollinearity was checked by variance inflation factor (VIF), and no multicollinearity exists between variables. The model fitness of test was checked by the Nagelkerke *R*^**2**^ goodness of the fit (*p*-value = 0.117).

## 3. Results

### 3.1. Sociodemographic Characteristics

A total of 364 students were participated in the study, making a response rate 99.7%. The majority (309; 84.9%) adolescents were found in the age range of 14–17 years. A little over half (50.8%) of the respondents were males. Regarding the current educational levels of their mothers, 72 (19.7%) of them had no formal education. Nearly, three fourth (230; 63.2%) of the respondents had a family size of > 5 (Table 1).

### 3.2. Water, Sanitation, and Hygiene-Related Factors

All of the respondents were using an improved source of drinking water. Almost all (360; 98.9%) of the study subjects had latrine in their home, and 358 (98.4%) were utilizing it. More than two thirds (252; 69.2%) of the respondents had a hand-washing facility near by the toilet, and 184 (50.5%) respondents washed their hands at critical times.

### 3.3. Dietary- and Life-Style-Related Factors

Only 95 (26.1%) adolescents did physical activity. Of the 95, 71 (20.9%) and 12 (3.3%) performed a vigorous and moderate-intensity physical activity, respectively. More than half (210; 57.7%) of the respondents did not use any transport systems to go to school.

In the past two weeks, 212 (58.2%) adolescents visited health facilities. However, only 12 (3.3%) received nutritional services like nutrition counseling, education, iron folate supplementation, and deworming. The majority (339; 93.1%) consumed a meal ≥3 times per day. More than half (206; 56.6%) of the adolescents skipped at least one meal within 24 hours. One hundred fifty-four (42.3%) had low dietary diversity (DD). Only 18 (4.9%) respondents drank alcohol within 24 hours.

### 3.4. Nutritional Status of High School Adolescents

**Figure 1:**
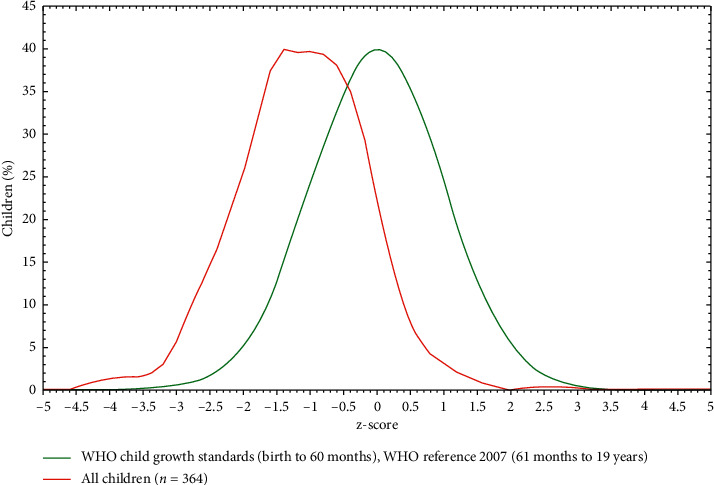
Height for age Z score of adolescent students of Dessie town high school, 2018.

The overall prevalence of stunting among high school adolescent was 15.7%.The prevalence of stunting was higher in males than females (Figure 1).

**Figure 2:**
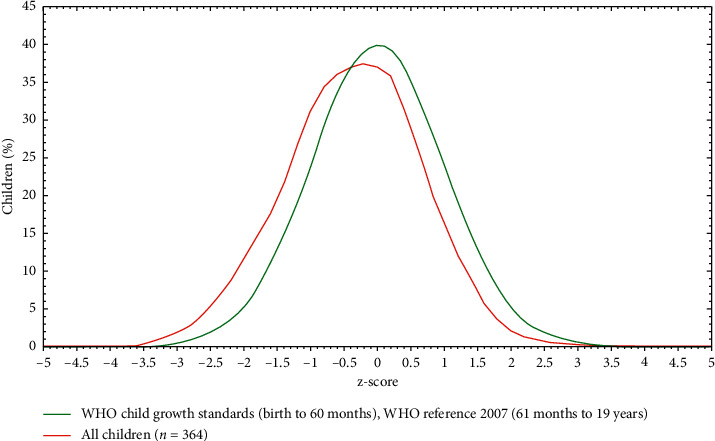
BMI for age Z score of adolescent students, Dessie town high school, 2018.

The magnitude of underweight and overweight/obesity was 6.3% and 8.2%, respectively (Figure 2). The prevalence of overweight/obesity was higher in females than males, but in the case of undernutrition, it is the opposite.

### 3.5. Factors Associated with Malnutrition among High School Adolescents

The odds of being stunted among high school adolescents who consumed snacks daily was 62% less common compared with those who did not consume snack daily (AOR = 0.38,95% CI: 0.20, 0.71). High school adolescents within adequate mean adequacy ratio were almost three times more likely to be stunted compared with those who had adequate food quality (AOR = 3.36, 95% CI: 1.15, 7.82) (Table 2).

The result of multinomial logistic regression model revealed that sex of adolescent, meal pattern, dietary diversity, and mean adequacy ratio were factors associated with underweight and overweight/obesity of adolescents. Male adolescents were almost three times more likely to be underweight/thin compared with female adolescents (AOR = 2.76, 95% CI: 1.03, 7.44); adolescents who consume a meal <3 times per day were four times more likely to be affected by underweight/thinness compared with adolescents who have ≥3 meals per day (AOR = 4.21, 95% CI: 1.35, 13.11) (Table 3).

Adolescents with poor dietary diversity were almost 65% less likely to become overweight/obese compared with those with adequate dietary diversity (AOR = 0.35, 95% CI: 0.13, 0.89). Adolescents with inadequate diet quality (MAR<1) were three times more likely to become overweight/obese compared with their counterparts (AOR = 3.14, 95% CI: 1.09, 9.09) (Table 3).

## 4. Discussion

The purpose of this study was to assess malnutrition and associated factors among school-going adolescents of Dessie town high schools. The result of this study indicated that the prevalence of stunting among adolescents was 15.7%. This is in line with studies done in Kenya 10.6% [17], West Bengal 18% [10], and Agarfa 12.2% [18]. However, this finding is higher than studies conducted in Addis Ababa (7.2%) [17] and Adwa [19], while it was lower than studies done in Timor-Leste (26.4%) [1] and Amhara region (31.5%) [16]. The possible reason for the variation in the prevalence of stunting could be due to socioeconomic difference and access to information on nutrition and feeding practice.

The percentage of underweight/thinness among adolescents was 6.3%. This is comparable with studies conducted in Addis Ababa (6.2%) [13] and Bahir Dar (8.6%) [19]. However, it was lower than studies conducted in Amhara region (13.6%) [16], Tigray Region (21.4%) [19], Chiro town (24.4%) [14], Hawassa (12.9%) [20], Timor-Leste (49.2%) [1], Bangalore city (69.2%) [9], and West Bengal (11.3%) [10]. This variation could be due to cultural difference in dietary habit and caring practices.

The magnitude of overweight/obesity among adolescents was 8.2%. This finding is similar with studies conducted in Mekelle town (6%) [8], Addis Ababa (8.5%) [21], Agarfa (8.96%) [18], and Chiro town (4.1%) [14]. However, it is higher than studies conducted in Hawassa (2.8%) [20] and Banglore city (3%) [9]. In contrast, it was lower than the findings from Bahir Dar (16.7%) [22], West Bengal (11.4%) [10], and America (69%) [23]. The possible reasons for the difference might be due to socioeconomic difference, life style, and easily accessibility of junk and processed foods.

Snack consumption was found to be a significant factor for stunting. High school adolescents who eat snacks daily were almost 62% less likely to be stunted compared with adolescents who did not eat snacks daily. This finding is in line with the finding from Kenya [17]. The possible reason could be because frequent intake of snack increases intake of balanced diet/optimum nutrition which is important in promoting the normal growth of height in adolescents.

Another factor, which contributes for stunting among adolescent, was mean adequacy ratio. Adolescents who ate a poor-quality diet (MAR < 1) were nearly three times more likely to be stunted compared with adolescents who ate a good-quality diet. The possible explanation might be that an adequate supply of all essential nutrients has a paramount importance in satisfying the nutritional requirements for the maintenance of a body's growth, strength, physical work, cognitive ability, immunity, and good health. So, high school adolescents who consumed a low-quality diet below the recommended daily allowance could have a high vulnerability for short stature.

High school adolescents who consume less than three meals per day were almost three times more likely to be underweight/thin compared with adolescents who consume three or more meals per day. This finding is in line with the findings from Adwa [22], Kenya [17], Islamabad city, and Tamale Metropolis [24]. Low mean frequency is associated with inadequate intake of diet in terms of quality, quantity, and diversity. Since there is fastest growth during adolescent period and increased nutritional requirements, low frequency of meal intake will make them to become underweight/thin.

This study showed that thinness was significantly higher among boys than girls. Male adolescents were nearly three times more likely to be affected by thinness compared with females. This finding is in line with studies conducted in Mekelle [8], Chiro [14], and Kenya [17]. This might be because boys engaged in active life style including energy demanding activities compared with females, and since girls stay at home, they may have good access to food than males. This may increase their susceptibility for underweight/thinness. Studies from Bahir Dar [25] and Debre Tabor [26] revealed that sex has no difference of nutritional status of adolescents [2]. This may be due to the difference in cultural and educational level between the families of adolescents.

This study revealed that dietary diversity was associated with the development of overweight/obesity among adolescents. High school adolescents who had low dietary diversity score (DDS) were 65% less likely to develop overweight/obesity. This finding is similar with studies conducted in Iran [27], Sri Lanka [27], and Tehran [28], which stated that increased DDS was associated with the development of overweight/obesity. This could be because a decreased intake of dietary diversity is associated with a lower intake of total calories from fat, saturated fat, and cholesterol [29]. This will decrease the percentage consumption of most of the food groups and is associated with low weight gain. In addition, higher consumption of starchy vegetables such as peas, potatoes, and corn was associated with weight gain due to their higher glycemic load [30]. This will help to prevent the risk of developing overweight/obesity. Therefore, low DDS decreases the problem of overweight/obesity among adolescents. However, this study contradicts a study done in rural India [31]. This difference might be due to the type and proportion of foods consumed by study participants.

Based on the finding of this study, inadequate mean adequacy ratio was associated with the occurrence of overweight/obesity. High school adolescents who ate a diet of an inadequate quality were three times more likely to become overweight/obese. Intake of nutrients with a poor quality does not provide all the essential nutrients that the body needs for its normal function; rather, it contains foods with high energy content and high trans-fats [32]. Such kinds of diets are correlated with general obesity, abdominal adiposity, and other chronic diseases.

## 5. Conclusion

Stunting and underweight are mild public health problems, while the percent of overweight/obesity among adolescents in the study area is higher compared with the national and regional prevalence, and since it is higher than 5%, it is considered a public health concern. Sex, mean adequacy ratio, snack consumption, low dietary diversity, and frequency of meal consumption were factors associated with malnutrition. Therefore, increasing snack consumption, frequent consumption of meals, sex consideration, consumption of diversified diet, and improving overall diet quality should be incorporated in the prevention strategies of malnutrition among adolescents.

## Figures and Tables

**Table 1:** Sociodemographic characteristics of adolescents, Dessie town high school, 2018.

Variables	Category	Frequency	Percent (%)
Age	Early adolescence	11	3.0
	Middle adolescence	309	84.9
	Late adolescence	44	12.1
Sex of HH head	Male	304	82.7
	Female	63	17.3
Living with	Parents	326	89.6
	Others*∗*	38	10.4
Education level	Grade 9^th^	179	49.2
	Grade 10^th^	185	50.8
Parents marital status	Currently not married	84	23.1
	Currently married	280	76.9
Mother occupation	Trader	73	20.1
	Civil servant	92	25.3
	Housewife	189	51.9
	Others*∗∗*	10	2.7

*∗*Alone, grandparents, aunt, and uncle. *∗∗*Waver, daily laborer, and small scale enterprise.

**Table 2:** Factors associated with stunting among adolescents, Dessie town high school, 2018.

Variables	Stunting		COR 95% CI	AOR 95% CI
	Yes	No		
Sex				
Male	17	155	0.73 (0.40, 1.26)	
Female	6	159	1	

Snack frequency				
Daily	5	19	0.42 (0.22, 0.78)*∗*	0.38 (0.20, 0.71)*∗*
Not daily	18	295	1	1

Dietary diversity score				
Below mean	20	245	0.78 (0.45, 1.38)	
Above mean	3	69	1	

Mean adequacy ratio				
Adequate	21	266	1	1
Inadequate	2	48	2.89 (1.01, 8.33)	3.36 (1.15, 7.82)*∗∗*

*∗*Significant at *p* value <0.05. *∗∗*Significant at *p* value <0.01.

**Table 3:** Factors associated with BMI for age of adolescents, Dessie town high school, 2018.

Variables	Nutritional status			Corollary 95% CI	AOR 95% CI
	Underweight/thin	Normal	Overweight/obesity		
Sex					
Male	17	155	13	2.91 (1.12, 7.57)	2.76 (1.03, 7.44)*∗*
Female	6	159	14	1	1

Meal consumption					
< 3 times/day	5	19	1	4.31 (1.44, 12.88)*∗*	4.21 (1.35, 13.11)*∗*
≥3 times/day	18	295	26	1	1

Dietary diversity score					
Below mean	12	134	8	0.37 (0.15, 0.95)	0.35 (0.13,0.89)*∗∗*
Above mean	11	177	22	1	1

Mean adequacy ratio					
Adequate	21	266	18	1	1
Inadequate	2	48	9	2.77 (1.18, 6.53)	3.14 (1.09, 9.09)*∗∗*
Level of education					1
9^th^	7	158	14	0.43 (0.17, 1.08)	0.41 (0.16, 1.04)
10^th^	16	156	13	1	1

*∗*Factor for underweight, *∗∗*Factor for overweight/obesity. All are significant at *p* value <0.05.

## Data Availability

The data used to support the findings of this study are included within the article.

## Abbreviations

AOR: Adjusted odd ratio
BAZ: Body mass index for Age Z-score
BMI: Body mass index
DDS: Dietary diversity score
GPAQ: Global physical activity questionnaire
HAZ: Height for age Z-score
NNP: National nutrition program
NAR: Nutrient adequacy ratio
SD: Standard deviation
WHO: World Health Organization.
